# Magnetic Resonance Imaging/Transrectal Ultrasound (MRI/TRUS) Fusion-Guided Prostate Biopsy: A Single-Center Experience

**DOI:** 10.7759/cureus.83946

**Published:** 2025-05-12

**Authors:** San Yu Leung, Kiujing James Fung, Wai Hung Lester Shiu, Matthew Ka Ki Law, Alta Yee Tak Lai

**Affiliations:** 1 Department of Radiology, Pamela Youde Nethersole Eastern Hospital, Hong Kong, HKG

**Keywords:** biopsy, fusion, magnetic resonance imaging, prostate cancer, transrectal ultrasound

## Abstract

Introduction: The role of magnetic resonance imaging (MRI) targeted biopsy has increased in the diagnostic pathway of prostate cancer. The objective of this study was to describe our experience with MRI/transrectal ultrasound (MRI/TRUS) fusion-guided prostate biopsy in a regional hospital, with an emphasis on the technique of the procedure.

Methods: This was a single-center, retrospective study. Cases of MRI/TRUS fusion-guided prostate biopsy performed in Pamela Youde Nethersole Eastern Hospital, Hong Kong, from September 2022 to December 2024 were reviewed. Systematic biopsy was also performed for all cases. Data were retrieved from electronic medical records. Procedural-related technical factors, fusion success rate, complications, and histopathological results were reviewed.

Results: A total of 125 patients were included in the study, with successful MRI/TRUS fusion performed in 124 patients (99.2%). The median number of passes to each targeted lesion was three (range: two to five). A total of 29 targeted prostate lesions (12.2%, among 28 patients) showed adenocarcinoma. An additional 11 cases of adenocarcinoma were detected in the systematic biopsy cores. The overall per-patient prostate cancer detection rate was 31% (39/124). Post-biopsy complications were observed in 19 patients (15.3%), with acute retention of urine being the most common complication (n=10, 8.1%).

Conclusion: MRI/TRUS fusion-guided prostate biopsy is a feasible and safe procedure. Our experience with the procedure, including peri-procedure assessment, fusion technique, and biopsy technique, was described in detail. A combination of systematic and MRI/TRUS fusion-guided targeted biopsies of the prostate is recommended as part of the diagnostic pathway in prostate cancer.

## Introduction

In 2022, in Hong Kong, prostate cancer was the third-most-common cancer in men, accounting for 16.0% of all new cancer cases in males, and prostate cancer was the fourth leading cause of male cancer deaths [1]. Digital rectal examination and prostate-specific antigen (PSA) blood serum testing have been widely performed. Patients with a clinical suspicion of prostate cancer are traditionally referred for prostate tissue sampling by transrectal ultrasound (TRUS)-guided systematic biopsy, where tissues are sampled randomly at the apex, mid gland, and base of prostate on both sides, ranging from six to eighteen or more tissue cores [2]. However, as there is considerable overlap in TRUS appearance between prostate cancer and benign lesions [3], non-targeted systematic biopsy could lead to sampling error, especially in patients with large prostate size [4].

Since the introduction of magnetic resonance imaging (MRI) of the prostate in the 1980s [5], this imaging technique has been increasingly performed, and is now recognized as a useful and accurate modality in the detection and staging of prostate cancer [6]. Suspicious prostate lesions detected by MRI are subsequently sampled for tissue diagnosis. There are three common MRI targeted biopsy techniques: cognitive biopsy, MRI-ultrasound software fusion biopsy, and MRI in-bore guided biopsy, which are performed via a transrectal or transperineal approach [7]. 

The Department of Radiology at Pamela Youde Nethersole Eastern Hospital (PYNEH), a regional hospital in Hong Kong, has been performing MRI/TRUS software fusion-guided prostate biopsy since September 2022. To date, only limited studies regarding MRI targeted biopsy techniques have been performed in Hong Kong [8]. Therefore, the objective of this study was to describe our experience with MRI/TRUS fusion targeted prostate biopsy, with an emphasis on the biopsy technique of the procedure.

## Materials and methods

Study design

This was a single-center, retrospective study. All patients who were referred to the Department of Radiology, PYNEH, Hong Kong, for MRI/TRUS fusion-guided prostate biopsy from September 2022 to December 2024 were included in the study. Patient demographics, clinical information, radiological images, procedure records, biopsy-related technical factors, pathological findings, and subsequent outcomes on follow-up were reviewed.

This study was approved by the Hospital Authority Central Institutional Review Board (Central IRB) on 20 February 2025 (Ref No: CIRB-2025-056-2).

Study population

A total of 127 consecutive patients were referred for MRI/TRUS fusion-guided prostate biopsy. All patients had clinical suspicion of prostate cancer and had received multiparametric MRI (mpMRI) of the prostate, which was either performed in the public hospitals under the Hospital Authority, Hong Kong, or at private imaging centers. Suspicious lesions detected via MRI were scored using the Prostate Imaging Reporting and Data System (PI-RADS) version 2.1. Anatomical location was named according to PI-RADS atlas. All referred patients were diagnosed with suspicious prostate lesions with a PI-RADS score of three or above. Referred patients with contraindications to the procedure (i.e., presence of implantable electronic devices such as cardiac pacemakers) were excluded from the study.

Pre-procedural assessment and preparation

Procedure-associated bleeding risks were assessed based on the Society of Interventional Radiology Consensus Guideline for Periprocedural Management of Coagulation Status and Hemostasis Risk in Patients Undergoing Percutaneous Image-guided Interventions (SIR guideline). According to the SIR guideline, transrectal biopsy of the prostate is considered a high-bleeding-risk procedure involving pelvic and retroperitoneal organs [9]. Pre-biopsy blood tests including platelet count and clotting profile were performed. Medication history on anticoagulant or antiplatelet use was reviewed, and these drugs were withheld according to the SIR guideline. 

Prophylactic antibiotic therapy was given one hour before the procedure, with a regimen of 500 mg oral cefuroxime (a second-generation cephalosporin) and 500 mg oral levofloxacin (a fluoroquinolone). 

Indications, risks, and potential complications of the procedure were explained to the patients. Informed consent was obtained. Each patient was placed in the left lateral decubitus position, with hips and knees flexed.

Real-time transrectal ultrasound-MRI fusion

**Figure 1:**
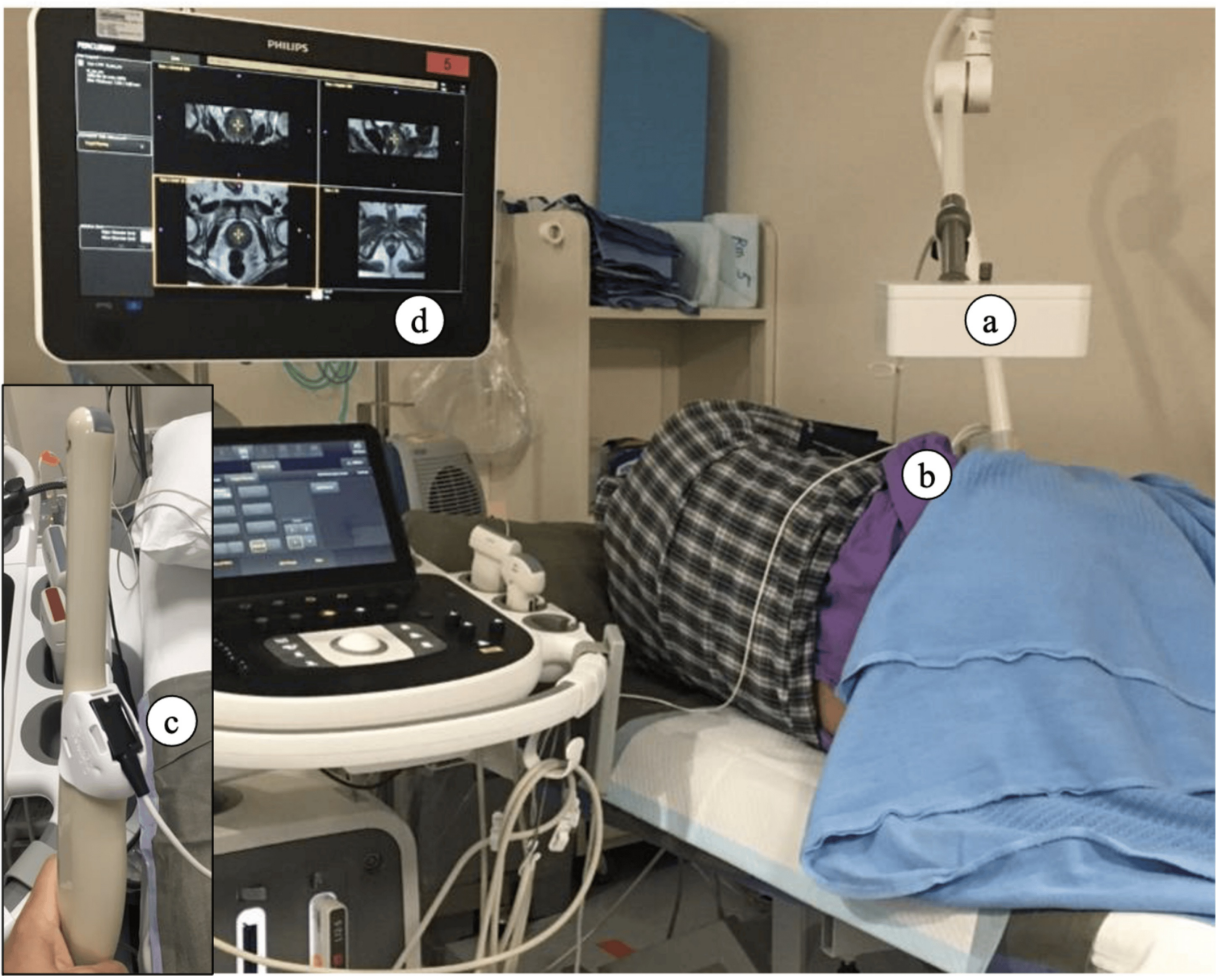
Electromagnetic tracking system setup (a) electromagnetic field generator; (b) patient tracker secured on the patient; (c) endocavity ultrasound tracker attached to the ultrasound transducer via a tracking bracket; (d) ultrasound machine with connection unit and MRI data uploaded. Abbreviations: MRI=magnetic resonance imaging

Each procedure was performed under ultrasound guidance using a 4-10 MHz curved endocavitary end-fire probe. Fusion of MR images with real-time ultrasound was performed using the PercuNav electromagnetic tracking system (Philips, the Netherlands), which consisted of an electromagnetic field generator, patient tracker, and endocavity ultrasound tracker (Figure 1). The electromagnetic field generator was positioned close to the patient (within 1 m). A patient tracker was secured on the patient’s hip or lateral upper-thigh region. An endocavity ultrasound tracker was attached to the ultrasound transducer via a tracking bracket. The electromagnetic field generator was used to create a position-varying magnetic field, inducing electric current in the ultrasound tracker. The position of the ultrasound transducer was varied to alter the magnitude of the electrical current. The position and orientation of the transducer was then calculated by the system, allowing tracking of the probe [10].

The MRI dataset was uploaded to the ultrasound system. T2-weighted oblique axial (perpendicular to the long axis of the prostate) and sagittal sequences (slices less than 3 mm) were used for fusion after co-registration. The targeted prostate lesions were selected.

**Figure 2:**
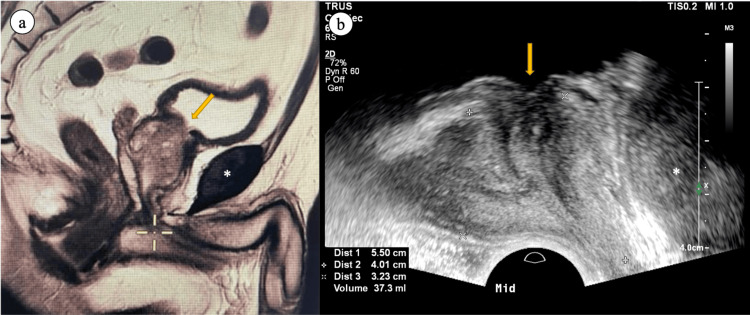
MRI/TRUS fusion using internal plane matching (a) midsagittal image of T2-weighted sagittal MRI sequence with orientation matched; (b) the same midsagittal plane under real-time ultrasound. The bladder neck (arrow) and the pubic symphysis (asterisk) were used as consistent landmarks to facilitate plane matching. Plane registration with fine adjustment was then performed. Abbreviations: MRI=magnetic resonance imaging; TRUS=transrectal ultrasound

After inserting the ultrasound probe, fusion of the real-time transrectal ultrasound and the MR images was performed using internal plane matching. The midsagittal image of the T2-weighted sagittal MRI sequence was selected while the prostate was scanned along the same midsagittal plane under real-time ultrasound. Plane registration was performed. Next, the bladder neck, a consistent landmark, was marked on both modalities for point registration. The MR image was rotated or tracked to match the real-time ultrasound image. Fine adjustment of fusion was performed by further point registration, using other landmarks such as the urethra or cysts (Figure 2).

**Figure 3:**
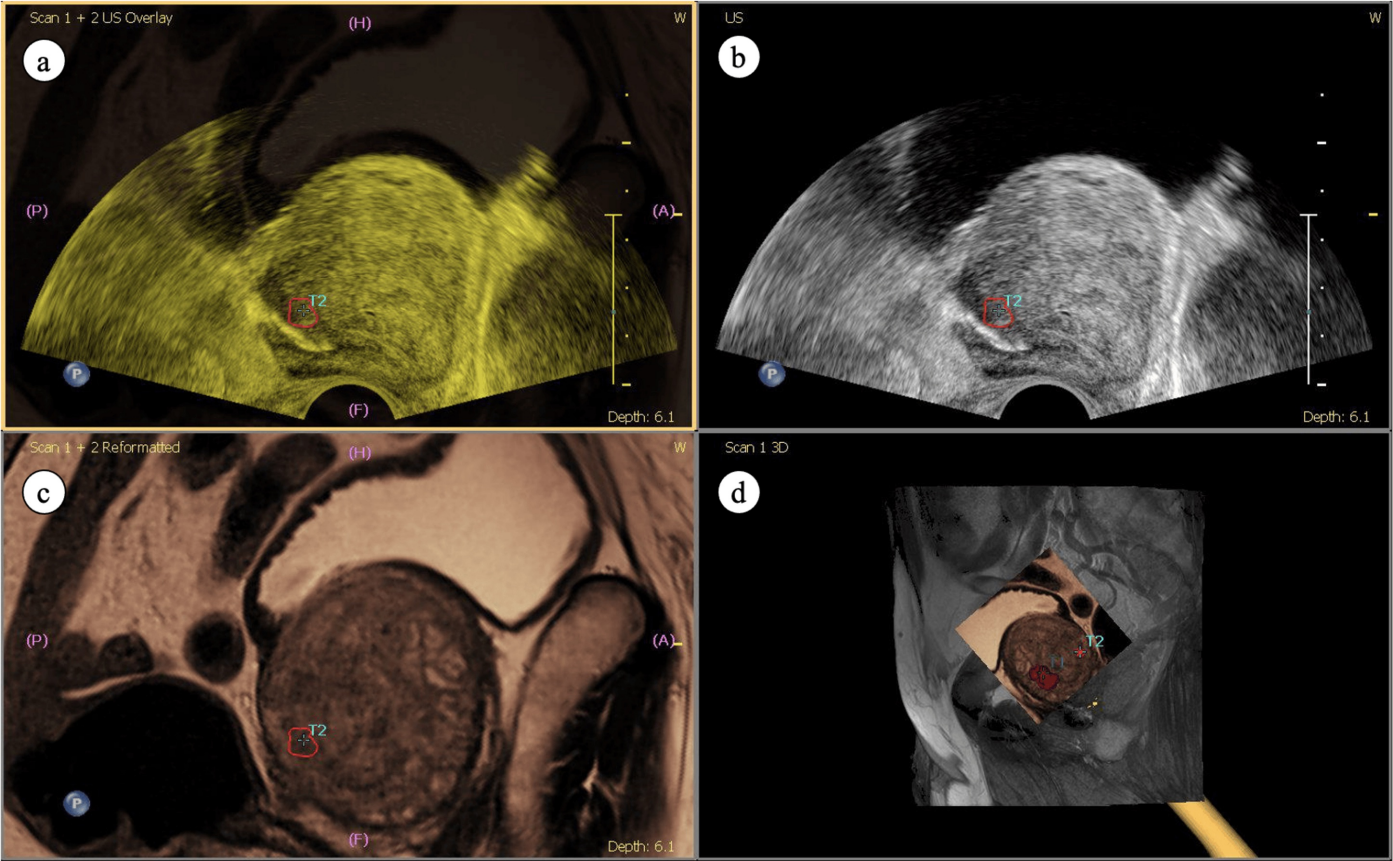
MRI/TRUS fusion images (a) blend overlay of both (b) ultrasound and (c) MR images, showing accurate fusion. The targeted lesion is indicated by the red marking. (d) Reconstructed three dimensional image according to real-time probe orientation. Abbreviations: MRI=magnetic resonance imaging; TRUS=transrectal ultrasound

After co-registration, the MR images were transformed into a projection that fit and superimposed the ultrasound image synchronously upon changing the position and plane of the ultrasound probe, which was deemed a technical success [11]. The targeted prostate lesions were then marked and localized on the fusion images (Figure 3).

Biopsy procedure and techniques

Prostate volume was measured using the ellipsoidal formula.

**Figure 4:**
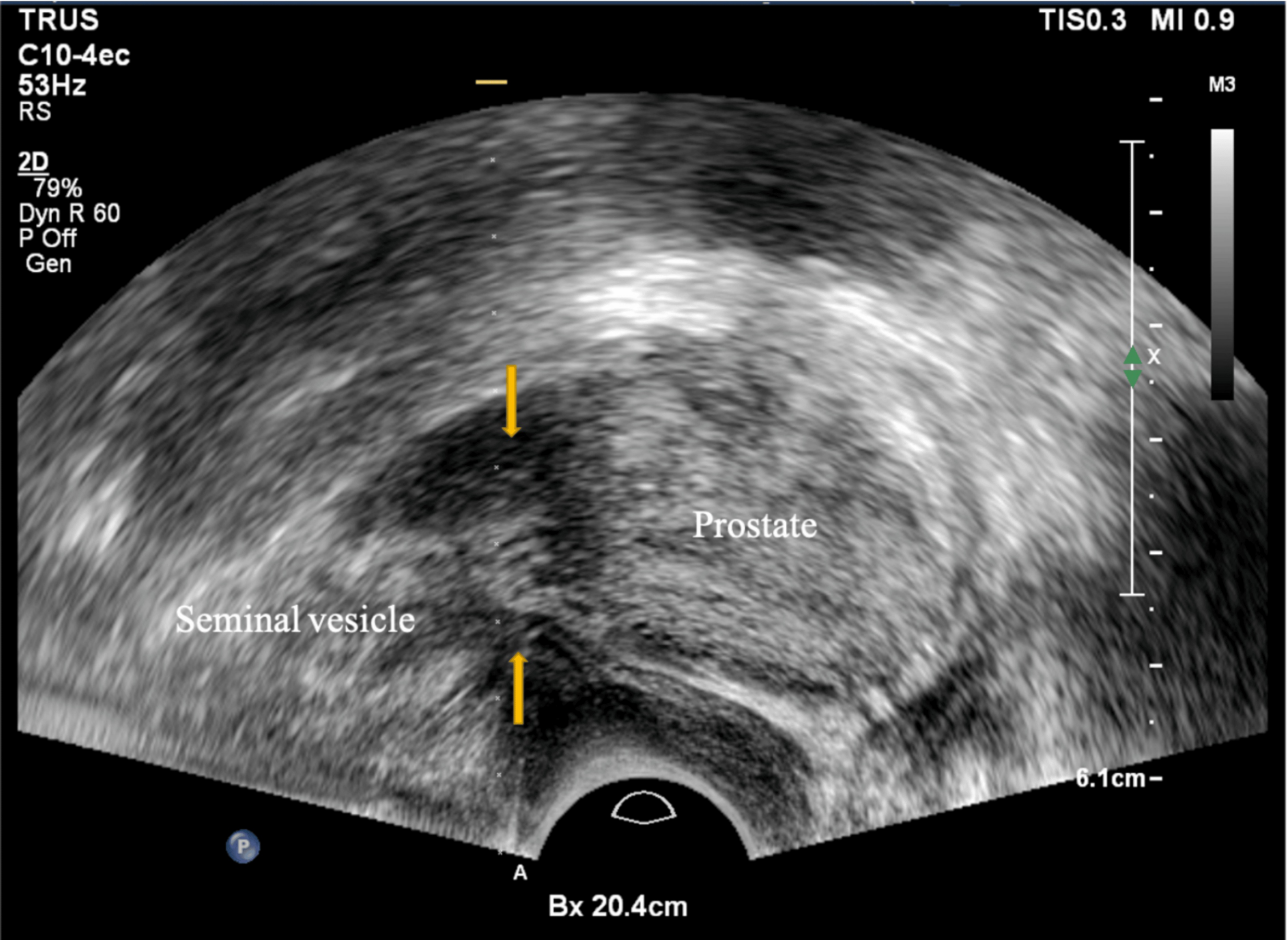
Site of PPNB Echogenic fat (arrows) at the junction between the prostate and seminal vesicles, where the prostatic vascular pedicle lies, was targeted for PPNB. Abbreviations: PPNB=peri-prostatic nerve block

A combination of intrarectal local anesthesia (IRLA) and peri-prostatic nerve block (PPNB) was applied for periprocedural pain management. 5 mL of 2% lidocaine gel was administered intrarectally 5 min before probe insertion as a topical anesthesia. PPNB was applied by injecting a total of 5 mL of 1% lidocaine at the rectoprostatic fascia bilaterally, in the region of the periprostatic neurovascular bundle at the junction between the prostate and seminal vesicles (Figure 4).

**Figure 5:**
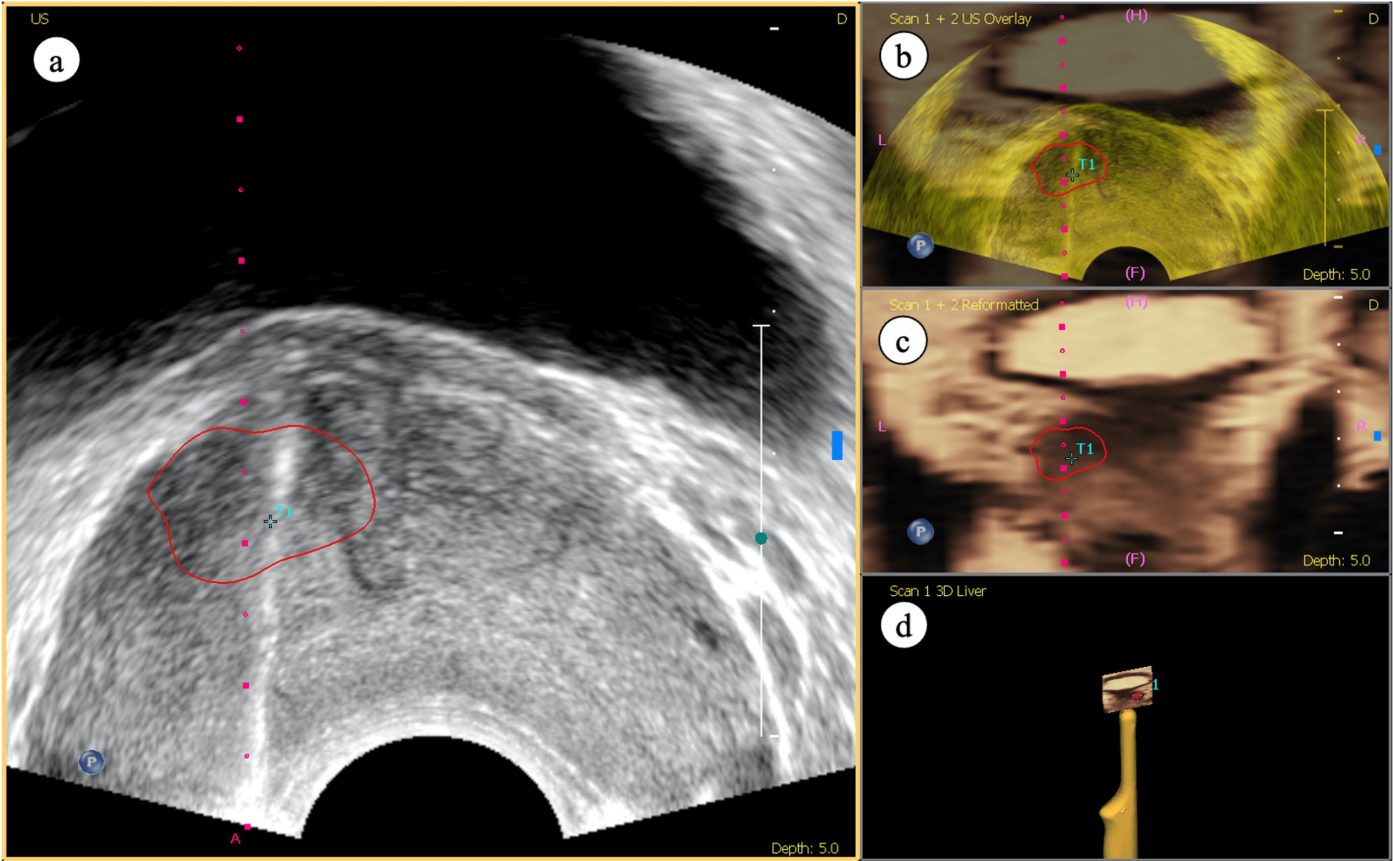
Biopsy of targeted prostate lesion under MRI/TRUS fusion guidance Biopsy needle is seen within the targeted lesion indicated by the red marking, as shown in (a) TRUS image, (b) blend overlay of both ultrasound and MR images, and (c) MR image; (d) reconstructed three dimensional image according to real-time probe orientation. Abbreviations: MRI=magnetic resonance imaging; TRUS=transrectal ultrasound

Biopsies were performed using an 18-gauge semi-automated biopsy needle with a 2-cm sampling notch and an end-firing needle guide. Targeted biopsies of the MRI-detected lesions were first performed under the guidance of real-time MRI/TRUS fusion (Figure 5). Subsequently, 12-core systematic biopsies were taken in the same session. 

An antibiotic regimen consisting of 500 mg oral cefuroxime twice a day and 500 mg oral levofloxacin once daily for three days was prescribed upon discharge.

Post-procedural assessment

Clinical records, including discharge summaries, emergency attendance notes, outpatient clinic consultation notes, and investigation results, were retrieved using the Electronic Patient Records, a territory-wide electronic medical record system shared by all public hospitals under the Hospital Authority. Complications within 30 days after biopsy were reviewed.

All histological results were traced via the same electronic medical record system.

## Results

Demographics, fusion success, and lesion characteristics

A total of 127 patients were referred for MRI/TRUS fusion-guided prostate biopsy. Two patients were excluded due to the presence of a pacemaker or an implantable cardiac defibrillator.

Successful MRI/TRUS fusion was achieved in 124 (99.2%) patients. Fusion failed in one (0.8%) patient due to a large prostate size (240 mL), resulting in poor penetration, limiting visualization of appropriate landmarks. In that case, cognitive fusion was performed instead.

Among the 124 patients, a total of 237 prostate lesions were biopsied under MRI/TRUS fusion guidance. The number of targeted lesions to be biopsied in each patient ranged from one to five, with the majority of patients having one (42%) or two (35%) lesions. The median number of passes to each targeted lesion was three (range: two to five), as seen in Table 1.

**Table 1:** Characteristics of patients (N=124) and targeted prostate lesions biopsied under MRI/TRUS fusion guidance (N=237). *Except otherwise specified. Abbreviations: MRI=magnetic resonance imaging; TRUS=transrectal ultrasound; PSA=prostate-specific antigen; mpMRI=multiparametric magnetic resonance imaging; HA=hospital authority; PYNEH=Pamela Youde Nethersole Eastern Hospital; PI-RADS=prostate imaging reporting and data system

Characteristic	n (%)*
Age (years), mean ± standard deviation(range)	69 ± 6 (48–83)
Prostate size (mL), mean ± standard deviation (range)	64 ± 26 (22–147)
PSA level (ng/mL), median (interquartile range)	10.1 (5.5–11.6)
PSA density (ng/mL^2^), median (interquartile range)	0.08 (0.05–0.11)
Centre where mpMRI of prostate was performed	
HA hospital (PYNEH)	44 (35)
HA hospital (non PYNEH)	4 (3)
Private sector	76 (61)
No. of targeted prostate lesions in each patient	
1	52 (42)
2	43 (35)
3	18 (15)
4	10 (8)
5	1 (1)
Reported PI-RADS score of targeted lesions	
1	0 (0)
2	0 (0)
3	102 (43)
4	107 (45)
5	28 (12)
Location of targeted lesions	
Transition zone	174 (73)
Peripheral zone	62 (26)
Central zone	0 (0)
Anterior fibromuscular stroma	1 (0.4)
No. of biopsy passes to each targeted prostate lesion	
2	17 (7)
3	186 (78)
4	31 (13)
5	3 (1)

Histology results

A total of 29 targeted prostate lesions (12.2%, among 28 patients) showed adenocarcinoma. Precancerous lesions of the prostate were found in four targeted lesions, with two showing high-grade prostatic intraepithelial neoplasia (HGPIN), and two showing atypical small acinar proliferation (ASAP), as seen in Table 2.

**Table 2:** Histopathological findings of targeted prostate biopsies under MRI/TRUS fusion guidance. Abbreviations: MRI=magnetic resonance imaging; TRUS=transrectal ultrasound.

Histopathological finding	No. (%) of lesions
Adenocarcinoma	29 (12)
High-grade prostatic intraepithelial neoplasia	2 (0.8)
Atypical small acinar proliferation	2 (0.8)
Scanty suspicious cell	1 (0.4)
Atypical glands	1 (0.4)
No evidence of malignancy	202 (85)

12-core systematic biopsies were taken in all cases, except one case where systematic biopsy was not possible due to prior extensive transurethral resection of the prostate (TURP). An additional 11 cases of adenocarcinoma were detected in the systematic biopsy cores, not being detected by targeted biopsies. On the other hand, three cases of adenocarcinoma were identified only in the targeted biopsy cores, with negative systematic biopsy results, as seen in Table 3.

**Table 3:** Distribution of adenocarcinoma-positive prostate biopsy cores. MRI=magnetic resonance imaging; TRUS=transrectal ultrasound.

Prostate biopsy cores	No. (%) of patients
MRI/TRUS fusion biopsy positive; systematic biopsy positive	25 (64)
MRI/TRUS fusion biopsy positive; systematic biopsy negative	3 (8)
MRI/TRUS fusion biopsy negative; systematic biopsy positive	11 (28)

The overall per-patient prostate cancer detection rate was 31% (39/124).

Associated complications

Post-biopsy complications within 30 days were observed in 19 (15.3%) patients, as seen in Table 4. Acute retention of urine was the most common complication (n=10, 8.1%). Most patients were able to wean off the urinary catheter within one week, except two who subsequently received TURP. There were two (1.6%) cases of hematuria, where one required hospitalization with bladder irrigation. Rectal bleeding occurred in four (3.2%) patients, among whom three required either rectal packing with adrenaline gauze or plication for hemostasis. One (0.8%) patient developed sepsis, requiring eight days of hospitalization.

**Table 4:** Complications after prostate biopsies within 30 days.

Complication	No. (%) of patients
Acute retention of urine	10 (8.1)
Hematuria	2 (1.6)
Rectal bleeding	4 (3.2)
Sepsis	1 (0.8)
Abdominal pain	1 (0.8)
Dysuria	1 (0.8)

## Discussion

With the accelerated application of MRI in the diagnostic pathway of prostate cancer, the number of suspicious prostate lesions detected by MRI is increasing, leading to a surge in demand for tissue diagnosis of these lesions. Traditional use of grayscale imaging in ultrasound lacks sufficient sensitivity to detect prostate lesions [3], leading to the development of strategies for MRI-targeted biopsies. Three common MRI-targeted biopsy techniques include cognitive biopsy, MRI-ultrasound fusion biopsy, and MRI in-bore guided biopsy. 

Cognitive fusion requires the operator to mentally co-register the location of MRI-detected lesions with ultrasound images. Tsang et al. from another local institution evaluated the efficacy of cognitive-fusion MRI/TRUS prostate biopsy and reported the advantage of its ready availability without requiring significant extra resources [12]. However, this technique demands a high level of expertise in terms of mental co-registration with MRI images. 

MRI in-bore guided biopsy allows direct visualization of the suspicious prostate lesions, which can increase operator confidence. However, the patient experience can be suboptimal due to the long procedural time within the MRI gantry, during which the patient remains in an uncomfortable prone position [2]. A recent meta-analysis showed no significant differences in prostate cancer detection rate among the above-mentioned three biopsy techniques [13]. The choice of these techniques may depend on resource accessibility, procedural time, and operator preference.

MRI/TRUS fusion technique

**Figure 6:**
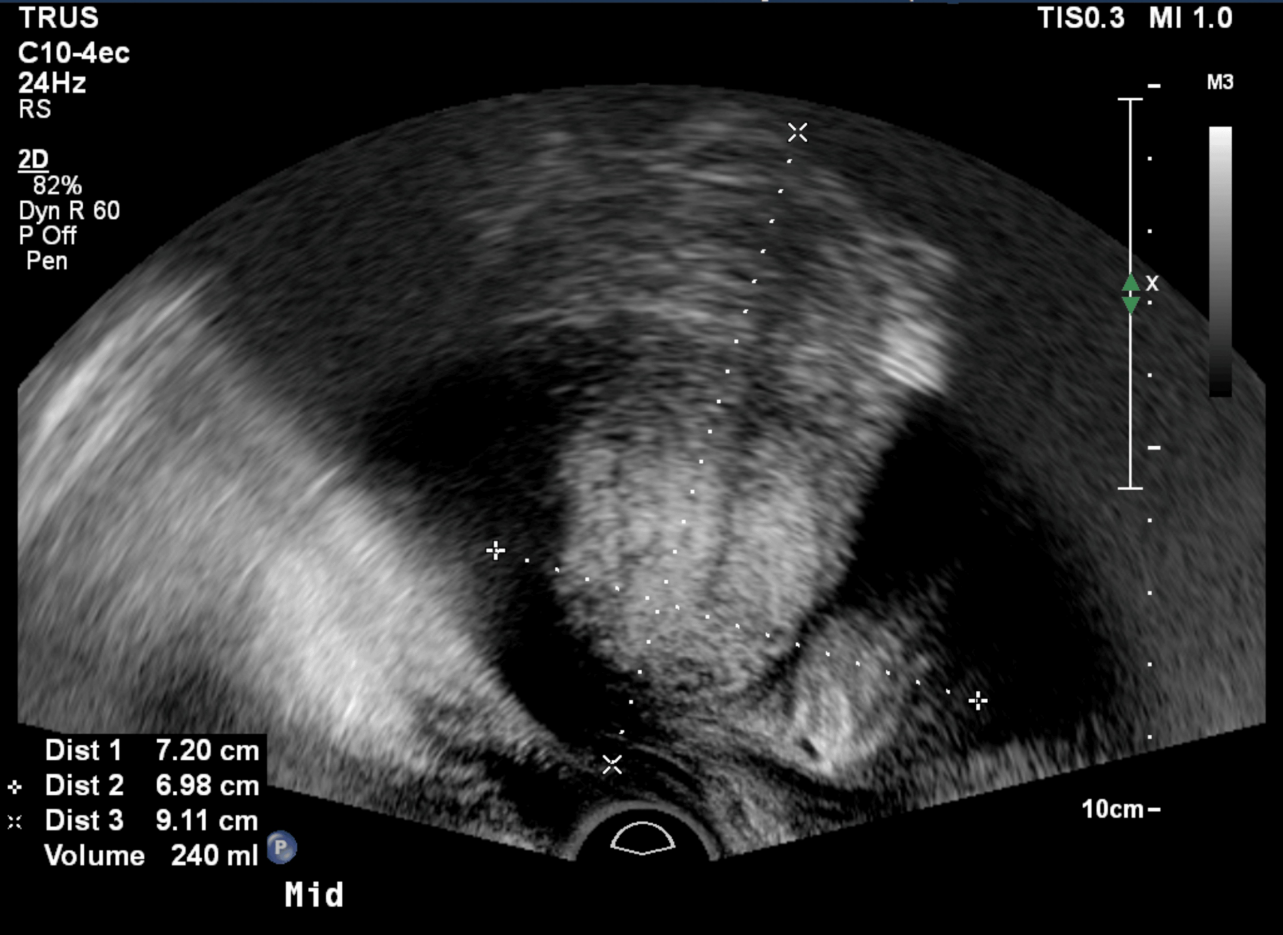
Case of failed fusion Large prostate size exceeded the sonographic field of view with poor penetration, limiting visualization of appropriate landmarks for fusion.

MRI/TRUS fusion of the prostate is a feasible procedure with a high success rate. In this study, we achieved technical success in 99.2% of patients. The only case where fusion failed was due to large prostate size, which exceeded the sonographic field of view with poor penetration, limiting visualization of appropriate landmarks for fusion (Figure 6).

The majority of patients are eligible for MRI/TRUS fusion, with the only contraindication being the presence of a pacemaker or automatic implantable cardiac defibrillator. The magnetic field induced by the electromagnetic field generator may interfere with the proper function of such cardiac devices, posing potential harm. Therefore, the use of these cardiac devices should be included in the pre-procedural checklist to optimize patient safety.

T2-weighted MRI images of 3-mm slice thickness are preferred for optimal resolution and landmark discrimination. Axial images are best for target lesion localization, whereas sagittal images allow for optimal plane registration.

There are two methods of MRI/TRUS fusion of the prostate: internal plane registration and internal landmark matching. Internal plane registration matches the same plane in both modalities, as described previously. Internal landmark matching refers to point registration using three points. Three anatomical landmarks, such as the urethra, calcifications, and cysts, are marked on both ultrasound and MR images. Fusion accuracy is verified by scanning various planes. The process can be repeated until optimal registration is obtained. In our experience, internal plane registration was the preferred method. The midsagittal plane of the prostate is easily reproducible in both ultrasound and MRI, with consistent landmarks in most patients, allowing simple and quick fusion. In contrast, internal landmark matching is often difficult and time-consuming, as anatomical landmarks that are easily identified on both imaging modalities are not always present. 

A point to note when performing MRI/TRUS fusion is the pressure applied to the transducer. Too much or too little transducer pressure can change the shape and position of the prostate, resulting in image distortion that does not match the MR images, posing a risk of inaccurate biopsy. Therefore, the operator should attempt to maintain a constant transducer pressure during the entire procedure and avoid any image misregistration. Software allowing co-registration with automatic motion compensation could re-register the images and minimize such error [2].

Biopsy technique

Transrectal biopsy of the prostate involves the needle passing through the rectal wall, potentially introducing fecal material into the urinary tract and the nearby bloodstream. Prophylactic antibiotic therapy was routinely prescribed to all patients to reduce infection risk, which was shown to be effective [14,15].

Pain management is an important yet sometimes overlooked aspect in all interventional procedures. Suboptimal periprocedural pain control could negatively affect the patient’s experience and complicate a procedure. During transrectal prostate biopsy, pain is encountered during probe manipulation, local anesthetic injection, and biopsy. We adopted a combination of IRLA and PPNB, as recommended by various international guidelines, such as those of the National Comprehensive Cancer Network (NCCN), the European Association of Urology (EAU), and the Korean Society of Urogenital Radiology (KSUR) [16-18]. The working principle of this approach is based on the nerve supply of the prostate and the anal canal. IRLA helps to reduce somatic pain from the anal canal and prostatic apex, which are innervated by the pudendal nerve, while PPNB numbs the pre-sacral and hypogastric nerve plexus, which innervate the prostatic capsule and parenchyma.

The optimal number of biopsy cores for targeted prostate lesions remains to be determined. One literature review showed that more clinically significant prostate cancers were detected when the number of biopsy cores per lesion was increased up to five [19]. However, more biopsy passes might increase the risk of complications [19], particularly in cases with multiple targets. Therefore, a case-by-case approach might be more useful in determining the appropriate number of biopsy cores. In our practice, the number of biopsy cores was determined by the operator, with three cores taken in the majority (78%) of cases, and a minimum of two and up to five cores taken depending on the number of targeted lesions.

Cancer detection

In a prospective study, MRI/TRUS fusion biopsy in conjunction with systematic biopsy showed an overall per-patient prostate cancer detection rate of 54.4% [20]. In our study, we achieved a lower overall per-patient prostate cancer detection rate of 31% (39/124). This difference is likely related to the presence of uncontrolled confounding factors. Cancer detection rate could be affected by patient selection. As the primary aim of this study was to discuss the biopsy technique used, all patients who underwent the procedure were included. The diagnostic mpMRI of these patients was performed in various centers, with possible variations in image quality and technique. Lesion characterization and grading might also be subject to variance among different reporting radiologists. 

Despite the above-mentioned limitations, the histopathological results in our study could provide support in our current practice of performing both targeted and systematic prostate biopsies. Three cases of adenocarcinoma were identified in the targeted biopsy cores, but not in the systematic biopsy cores. All three patients were later categorized as high risk after considering PSA level, Gleason score and grade group, and the clinical stage. These results illustrate the importance of targeted biopsy in achieving accurate and timely diagnosis.

On the other hand, there were 11 cases of prostate cancer undetected by targeted biopsy but instead found in systematic biopsy cores. The additional disease detection rate (28%) was higher than that described in the literature [21], partly due to the heterogeneity in patient selection. Nonetheless, this result emphasizes the role and added value of concomitant systematic biopsy in the diagnostic pathway. 

There has been debate on the best biopsy pathway (e.g., targeted biopsy alone versus a combination of targeted and systematic biopsy) in patients with intermediate and high likelihood of prostate cancer. The diagnostic accuracy of clinically significant cancers, overdiagnoses of clinically insignificant cancers, and biopsy-related morbidity are all under consideration. Generally, guidelines from the NCCN and the EAU recommend a combined approach of prostate biopsy [16,17], which remains preferable based on our experience.

Complications

The MRI/TRUS fusion process itself is non-invasive, with no concern of causing major complications. Improper application in contraindicated patients (i.e., those with a cardiac device) may lead to device malfunction, resulting in improper pacing and trigger shocks. Although it is inevitable that MRI/TRUS fusion increases procedural time, the additional time spent varies depending on the level of experience of the operators [10]. In our practice, three to four cases of MRI/TRUS fusion-guided prostate biopsy are scheduled in each biopsy session, which is similar to that in a systematic TRUS-guided prostate biopsy session. Increased operator experience and support from specialists/sonographers specialized in fusion imaging could minimize the additional procedural time. 

The possible complications encountered during biopsy are equivalent to those in systematic TRUS-guided prostate biopsy. The number of cores taken could affect the rate of complications [22]. As both targeted and systematic prostate biopsies are performed, more biopsy cores are taken. In Hong Kong, there have been few reports of complications resulting from TRUS biopsy. Cheng et al. conducted a five-year retrospective review of complications of TRUS biopsies in two local hospitals [23]; we reported a similarly low rate of post-biopsy complications. Future studies comparing the complication rate of targeted plus systematic biopsy versus one technique alone could provide more insight.

The transperineal approach has been gaining popularity in view of its lower risk of infection and rectal injury/bleeding compared to the transrectal approach [24]. Transperineal MRI US fusion targeted prostate biopsy is now a well-established technique and will definitely play a more significant role in our future practice.

Limitations

This study had several limitations. First, the primary aim of this study was to share our experience in MRI/TRUS fusion targeted prostate biopsy, with an emphasis on the technique used. The histopathology results were mainly descriptive. Statistical analysis and comparison were not carried out due to the presence of uncontrolled confounding factors, as mentioned previously. Second, we did not stratify the proven cases of prostate cancer by their clinical significance (i.e., Gleason score or grade group) because management also depends on clinical factors (i.e., PSA level and staging), and any positive pathology, including grade group one, can be important. Third, non-recorded complications such as emergency attendances or hospitalizations to the private sector were not included.

## Conclusions

MRI/TRUS fusion-guided prostate biopsy is a feasible and safe procedure. Our experience with the procedure, including peri-procedure assessment, fusion technique, and biopsy technique, were described in detail. A combination of systematic and MRI/TRUS fusion-guided targeted biopsies of the prostate is recommended as part of the diagnostic pathway in prostate cancer.
